# Sex and Age-Group Differences in Strength, Jump, Speed, Flexibility, and Endurance Performances of Swedish Elite Gymnasts Competing in TeamGym

**DOI:** 10.3389/fspor.2021.653503

**Published:** 2021-05-13

**Authors:** Stefan Höög, Erik P. Andersson

**Affiliations:** ^1^Department of Health Sciences, Swedish Winter Sports Research Centre, Mid Sweden University, Östersund, Sweden; ^2^School of Sport Sciences, Faculty of Health Sciences, UiT the Arctic University of Norway, Tromsø, Norway

**Keywords:** athletic performance, code of points, gymnastics, muscle strength, physical fitness, testing, gender

## Abstract

**Purpose:** To analyze sex and age group differences in strength, jump, speed, flexibility, and endurance performances of TeamGym athletes.

**Methods:** A total of 91 Swedish elite gymnasts (junior female, *n* = 26, age = 15.4 y; senior female, *n* = 23, age = 20.0 y; junior male, *n* = 19, age = 15.6 y; senior male, *n* = 23, age = 20.6 y) participated in three testing sessions on three separate days. These were: (1) a series of flexibility tests for the lower- and upper-body; (2) strength tests for the lower- and upper-body; and (3) various types of jumps, a 20-m sprint-run, and a 3,000-m run test.

**Results:** Males were 24% stronger in the back squat one-repetition maximum (relative to body mass) compared to females (*P* < 0.001, *H*_*g*_ = 1.35). In the pull-ups and dips, 2.4 and 2.3 times more repetitions were completed by the males compared to the females (both *P* < 0.001, 0.70 ≤ *R* ≤ 0.77). However, females were similarly strong as males in the hanging sit-ups test (*P* = 0.724). The males jumped 29, 34, 33, and 17% higher in the squat jump (SJ), countermovement jump (CMJ), countermovement jump with arm swing (CMJa), and drop jump (DJ), respectively, compared to the females (all *P* ≤ 0.002, 0.14 ≤ $\eta_{p}^{2}\;\leq$ 0.60). In the 20-m sprint run, males were 4% faster than females (*P* < 0.001, *R* = 0.40). Moreover, the females had significantly better flexibility than the males in the trunk forward bending, front split, and side split tests (all *P* < 0.001, 0.24 ≤ $\eta_{p}^{2}$ ≤ 0.54). In the 3,000-m run test, males were 11% faster than females (*P* < 0.001, $\eta_{p}^{2}$ ≤ 0.54). Compared to junior athletes, seniors performed better in the pull-ups, dips, SJ, CMJ, CMJa, and 20-m sprint-run tests (all *P* ≤ 0.012, 0.31 ≤ *R* ≤ 0.56, 0.16 ≤ $\eta_{p}^{2}$ ≤ 0.25), with separate within-sex age-group differences (i.e., juniors vs. seniors) that were significant for the males but not for the females in the SJ, CMJ, CMJa, and 20-m sprint-run tests (males: all *P* < 0.001, 0.67 ≤ *R* ≤ 0.69, 1.37 ≤ *H*_*g*_ ≤ 2.01; females: all *P* = 0.298–732).

**Conclusions:** Large sex and age-group differences were observed for most physical performance metrics with specific within-sex age-group differences only observed for male athletes, with male seniors performing better than juniors in the SJ, CMJ, CMJa, and 20-m sprint-run tests.

## Introduction

Gymnastics is a sport that combines characteristics of strength, explosive power, speed, and flexibility (Bale and Goodway, 1990; Bencke et al., 2002; Jemni et al., 2006) and is governed by the International Gymnastics Federation (FIG). One relatively new discipline of gymnastics is TeamGym, where a team of 8-10 participants competes in three apparatuses – the floor, tumbling, and trampette. Briefly, the TeamGym floor program involves choreography to music lasting between 2:15–2:45 min:s and includes flexibility movements, jumps, acrobatics, and balance elements. The tumbling routine comprises a tumbling track with a run-up of 16 m where the gymnasts perform series of acrobatic elements backward and forwards. The trampette routine uses a square-formed mini-trampoline with a 25-m run-up where the gymnasts perform somersaults with and without a vaulting table. All three routines require, in addition to physical fitness, high technical skills in acrobatic and gymnastic elements. A combined final score of the floor program and the three different rounds in the tumbling and trampette routines rank the team, applying the rules defined in the Code of Points which is reviewed and updated every four years (Sjöstrand et al., 2017). The performance level of TeamGym has increased rapidly based on the gradually enhanced difficulties from the first official European Championships in 2010 until the present day. This was likely related to the introduction of the 2010 years version of the Code of Points ranking system (Hughes et al., 2010) that was based on a new open-ended difficulty score, which has led to faster development of more advanced gymnastic elements. In addition, the regular updates of the Code of Points ranking system (every 4 years) have likely also had an impact on the performance characteristics of the sport. All these factors have possibly contributed to the increased physical and technical demands of elite-level TeamGym athletes.

FIG has testing and training programs for the other disciplines of gymnastics such as the age group development program for female artistic gymnastics for gradual evaluation of physical capacities and athletic development over time (Fink et al., 2015). Another testing program for artistic gymnastics is the functional measurement tool (Sleeper et al., 2012, 2016). However, there are no specifically developed testing and training programs for the TeamGym discipline. A TeamGym athlete is likely to benefit from having high explosive strength and maximal strength capacity especially in the lower body, which is very useful for rebounding from the floor and for vaulting during various types of somersaults (Hansen et al., 2019). Since a high run-up speed was found to be important for the performance score during various vaults in artistic gymnastics (Schärer et al., 2019), a high run-up speed is also likely to be related to the performance of the somersaults performed in TeamGym. As based on the performance characteristics of TeamGym and the data presented by Hansen et al. (2019), a test battery involving various maximal jumps, sprint running, and maximal strength tests might be of relevance for both physical profiling, as well as regular testing, of TeamGym athletes. Although the popularity of TeamGym is growing fast in Europe, research on this sport is still sparse, with most previous studies only addressing injury incidence/symptoms (Harringe et al., 2004, 2007; Lund and Myklebust, 2011). Currently, there is only one study that has detailed the physical fitness of senior TeamGym athletes where lower-body muscle function was evaluated using tests of vertical jump, linear sprint performance, and isometric leg press (Hansen et al., 2019). The results from that study showed moderate associations between mechanical lower-body muscle function and tumbling performance, as well as significant sex differences for almost all physical capacities.

Although the Code of Points has identical difficulty values for all elements of the routines in TeamGym for males and females, it is well known that males are taller and heavier than females and that males have greater maximal strength, explosive strength, sprinting speed, and endurance characteristics (Kraemer et al., 1989), while females are noticeably more flexible than males (Bale et al., 1992). Compared to females, males experience a more substantial increase in body mass and strength during the final years of adolescence, which may impact some physical abilities differently in the transition from junior to senior age (e.g., from an age of 15 to 20 years) in males vs. females (Kraemer et al., 1989; Handelsman, 2017). Moreover, sex differences in sports performance increase gradually after the age of 12–13 years and reach a plateau after the age of ~20 years, which has been related to the rise in circulating testosterone due to puberty (Handelsman, 2017; Handelsman et al., 2018). The effect of pubertal age on sex-differences has also been shown to be relatively consistent for various types of physical abilities such as sprint running, middle-distance running, swimming, and handgrip strength (Handelsman, 2017). Maximal sprint ability is of substantial importance to performance in many sports with considerable sex differences commonly observed in senior athletes (Hansen et al., 2019; Nuell et al., 2019; Cardoso de Araújo et al., 2020). For instance, sex differences in sprint running have been attributed to the disparity in force/power and muscle volume characteristics (Nuell et al., 2019). In the study by Nuell et al. (2019), male senior sprinters had larger leg muscle volumes (especially in the hamstring muscle) and greater sprint mechanical properties than female sprinters which contributed to a 15% faster 80-m sprint time. Moreover, Askow et al. (2019) showed in a group of resistance-trained males and females (~21 years of age), that males back squatted 30% more weight per kilogram of body mass than females. In a group of German Bundesliga soccer players, males were 11% faster in a 20-m linear sprint-run test and jumped ~45% higher in the squat jump (SJ) and countermovement jump (CMJ) tests, when compared to females (Cardoso de Araújo et al., 2020). In a study conducted on Danish elite TeamGym athletes, Hansen et al. (2019) reported males to sprint 9% faster in a 25-m linear sprint-run test and to jump 24% higher in a CMJ test.

To date, there is no data available regarding sport-specific strength, jump, speed, flexibility, and endurance capabilities of elite-level TeamGym athletes and there is currently no data on how these abilities are characterized in different sex and age groups. Such information would be useful for optimizing training for athletic performance and to provide vital information for further updates to the Code of Points ranking system. Therefore, this study aimed to report the physical capacities of the best Swedish male and female TeamGym athletes at junior and senior levels as well as to analyze the effect of sex and age group (i.e., junior vs. senior athletes) on physical capacities. Based on a previous study by Hansen et al. (2019), we hypothesized that the senior males would jump ~24% higher in the CMJ and run ~9% faster in the 20-m sprint-run test than the senior females and that these sex differences would be higher for senior athletes (age of ~20 years) than for junior athletes (age of ~15 years), as based on previous findings by Handelsman (2017).

## Methods

### Participants

Ninety-one elite-level gymnasts were recruited for this study including junior female (*n* = 26), senior female (*n* = 23), junior male (*n* = 19) and senior male (*n* = 23) athletes with the participant characteristics shown in Table 1. The study was performed according to the Declaration of Helsinki and had been pre-approved by the Swedish Ethical Review Board (#2019–06039). The inclusion criterion was that the athlete had been chosen for the first selection of the Swedish national team competing in TeamGym year 2020. The exclusion criteria were injuries and/or sickness that could affect the test results and/or pose a potential health risk for the athlete during testing. All participants received both written and verbal information about all the testing procedures and potential risks before they provided written informed consent.

**Table 1:** Participant characteristics for junior female (*n* = 26), senior female (*n* = 23), junior male (*n* = 19), and senior male (*n* = 23) TeamGym athletes presented as mean ± standard deviation (SD) with the exception for training time that is presented as median and interquartile range.

		**Females**	**Males**	**Combined**	**Test statistic**	***P*-value**	**ES**
Age (years)	J	15.4 ± 0.9	15.6 ± 0.8	15.5 ± 0.9	-	−	−
	S	20.0 ± 3.0	20.6 ± 3.2	20.3 ± 3.1	-	−	−
Body height (cm)	J	163.3 ± 5.8	174.3 ± 5.3	167.9 ± 7.8	^#^F_1,87_ = 109.0	<0.001	0.56
	S	163.8 ± 4.9	176.1 ± 5.1	170.0 ± 7.9	^$^F_1,87_ = 1.2	0.282	0.01
					^£^F_1,87_ = 0.3	0.561	0.00
BM (kg)	J	57.8 ± 6.3	65.7 ± 7.1^*§^	61.1 ± 7.7	^#^F_1,87_ = 69.0	<0.001	0.44
	S	59.4 ± 6.5	74.9 ± 6.9	67.2 ± 10.3	^$^F_1,87_ = 14.9	<0.001	0.15
					^£^F_1,87_ = 7.3	0.008	0.08
BMI (kg·m^−2^)	J	21.6 ± 1.5	21.6 ± 2.1^*§§^	21.6 ± 1.8	^#^F_1,87_ = 7.9	0.006	0.08
	S	22.1 ± 1.8	24.1 ± 1.3	23.1 ± 1.9	^$^F_1,87_ = 17.2	<0.001	0.16
					^£^F_1,87_ = 8.1	0.006	0.09
Training volume (h·week^−1^)	J	13 (12–15)	14 (12–16)	13 (12–15)	^#^U = 949	0.519	−0.07
	S	14 (14–15)	15 (14–17)	15 (14–16)	^$^U = 738	0.017	−0.25

*BM, body mass; BMI, body mass index; J, juniors; S, seniors. F-values, P-values, and effect sizes (ES), partial eta squared effect size ($\eta_{\text{p}}^{2}$), were obtained by a two-way ANOVA*.

# *Main effect for sex*.

$ *Main effect for age group*.

£ *Main effect for interaction between sex and age group. In case of a significant interaction effect, a within-sex-group comparison of J vs. S was performed with an independent t-test. For training time, U-values, P-values, and effect sizes (R) were obtained by a Mann-Whitney U-test*.

*§ *significantly different from male seniors (P < 0.001, Hedges' g effect size [H_g_] = −1.29)*.

*§§ *significantly different from male seniors (P < 0.001, H_g_ = −1.43)*.

### Study Design

The first testing session started with flexibility assessments that took ~20 min to complete for each participant with ~2 min of rest in between the different tests. The second testing session was strength exercises that took ~40 min to complete. This was followed by a third testing session including jumps, a 20-m linear sprint-run test, and a 3,000-m run test on a tartan track, which took ~1.5 h to complete for each participant. The three testing sessions were performed on three separate days interspersed by ~1 month between the first and second sessions and 4 days between the second and the third sessions. Before all testing sessions, the gymnast completed a 20-min structured warm-up and was informed to avoid hard physical activities 24 h before testing. The participants were well familiarized with all the different tests from previous years of training and testing.

### Equipment and Measurements

Upon arrival at the laboratory, the gymnasts were asked about their amount of sport-specific training, which included gymnastics and strength training, reported as an average over the latest training year and expressed as a weekly training volume (i.e., hours per week). Stature was measured by using a standard wall stick scale and reported to the nearest millimeter. Body mass was measured barefooted and in light clothing with a Beurer BG 19 scale (Beurer GmbH, Ulm, Germany). The range of motion during the flexibility tests was assessed by measuring specific distances (described in detail below) with a standard tape measure reported to the nearest millimeter or centimeter depending on the current recommendations for the specific test. The jumping performances of the SJ, CMJ, and CMJ including a free arm swing (CMJa) were determined with an Opto Jump Microgate system (Microgate, Bolzano, Italy), which calculates jump height based on the flight time in the air during a jump (Glatthorn et al., 2011). A piece of similar equipment (IVAR Jump and sprint system, Spin Test, Tallinn, Estonia) was used to measure the drop jump (DJ), and the 20-m linear sprint-run performance (Carlsson et al., 2012). For all jump and sprint-run tests, the gymnast performed three repetitions in each test with the best result being reported.

### Detailed Information About the Testing Procedure

The test battery was developed from former testing procedures in other disciplines of gymnastics and different sports with similar demands. The strength, jump, speed, and 3,000-m run tests were performed according to “Fysprofilen^1^”, which is a test battery developed by the Swedish Olympic Committee, that is frequently used for assessment of different qualities of physical performance in Swedish elite athletes. The flexibility tests were based on standard testing procedures that are commonly used in other gymnastic disciplines such as artistic gymnastics (Sleeper et al., 2012, 2016; Fink et al., 2015).

#### Flexibility Tests

##### The dorsiflexion lunge test for the right and left foot

**Figure 1:**
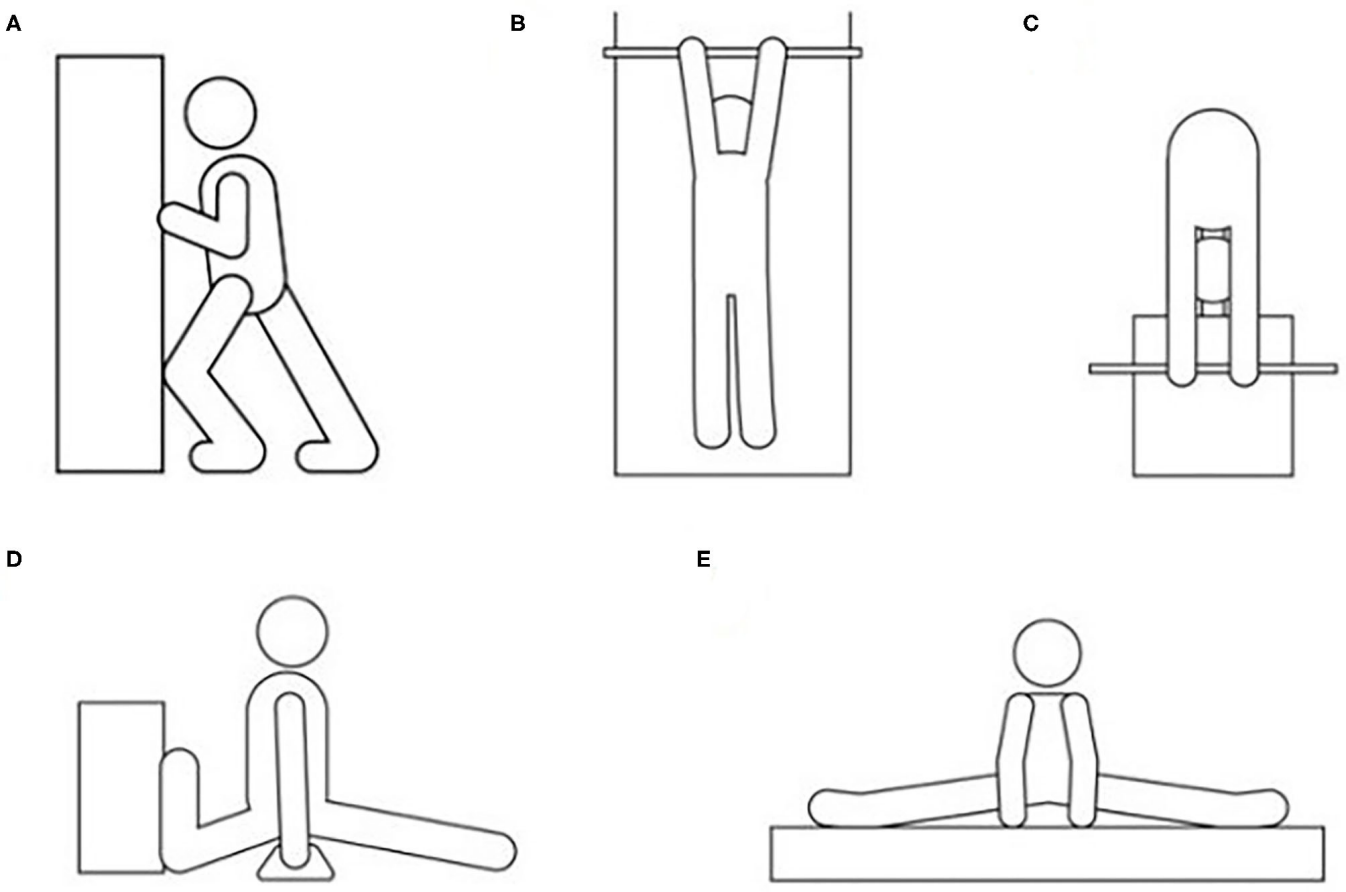
The figure describes how the flexibility tests were performed with: **(A)**, the dorsiflexion lunge test for the right and left foot; **(B)**, the shoulder flexibility test; **(C)**, the trunk forward bending test; **(D)**, the front split test for the right and left legs; and **(E)**, the side split test.

To assess ankle dorsiflexion, the participant placed one foot and both hands against a wall where after the knee was lunged toward the wall. The foot was then progressively moved away from the wall until the maximum range of dorsiflexion was reached (Figure 1A). During the lunge, the test leader held the heel to prevent it from lifting from the floor, with the knee in contact with the wall and with the tibia advancing over the talus into maximum dorsiflexion. The distance was measured from the wall to the participant's hallux with distance reported to the nearest millimeter.

##### The shoulder flexibility test

The shoulder flexibility was tested with the participant lying in a prone position with the stomach, chin, and nose in contact with the floor. Both arms were held straight and parallel to the body and flexed to 180°. The gymnast held a wooden dowel pin using an overhand grip with the wrist in a neutral position and with the thumbs touching each other where a maximal shoulder flexion with extended elbows was performed (Figure 1B). The distance was recorded from the floor to the dowel pin where the thumbs touched each other, to the nearest 0.5 cm (Sleeper et al., 2012). The distance was reported as positive (i.e., with more positive meaning more flexible).

##### The trunk forward bending test

The test was conducted with the participant standing on a bench with the feet together and with straight legs holding a wooden dowel pin by using an overhand grip while keeping the wrists in a neutral position and the thumbs touching each other. From this starting position, the participant bent forward and tried to come as close to the floor as possible (Figure 1C). The distance was recorded from the top of the bench to the dowel pin where the thumbs touched each other, to the nearest 0.5 cm. The distance under and over the bench was defined as positive and negative, respectively (Fink et al., 2015).

##### The front split test for the right and left leg

The participant performed the right front split with the left hip extended maximally and the right hip flexed maximally (vice versa for the left front split). The tibia of the left leg was placed against the wall vertically where after the participant was instructed to slide out into a split position. During the test, the back had to be vertically positioned to the hip with shoulders parallel to the wall (Figure 1D). To maintain a proper testing position, the participant was permitted to use floor parallel bars and with the test leader fixating the back foot for maintaining the tibia in a vertical position. The measurement of the distance was from the floor to the highest point of the perineal area and reported to the nearest 0.5 cm and reported as negative units (i.e., the less flexible the more negative score). If the participant maintained the split with full contact with the floor, the result was 0 cm (Sleeper et al., 2012).

##### The side split test

The participant started the test with the heels placed perpendicular to a straight line before performing a slide-out to a side split (both hips abducted maximally). At the same time, the participant leaned forward and placed both hands in front of the body (Figure 1E). The measurement was performed in front of the participant, from the floor to the highest point of the perineal area, and reported to the nearest 0.5 cm and in negative units. If the participant had full contact with the floor, the result was 0 cm (Sleeper et al., 2012).

All flexibility tests were conducted in the same order as reported in the text above and the participant had ~2 min of rest in between tests.

#### Strength Tests

##### The back-squat test

The purpose of this test was to evaluate the maximal strength of the lower body with the back squat exercise while measuring the maximum weight lifted once (1 RM) (Levinger et al., 2009). This test was a free weight exercise with a men's Olympic barbell (20 kg) placed on the shoulders behind the neck. From a standing position, the participant performed a controlled knee flexion until the thighs were parallel to the floor and then extended the knees back to the starting position. Each participant completed the tests with the following load increase: (1) five repetitions at 50% of estimated 1 RM; (2) three repetitions at 80% of estimated 1 RM; and (3) one repetition at 90% of estimated 1 RM. The result was presented as strength relative to body mass (i.e., total lifted weight [kg]/body mass [kg]). This test was only performed by the seniors (i.e., participants aged ≥ 18 years, or being 17 years turning 18 years within the specific year) as some of the younger junior athletes had no adequate experience of lifting technique training, and the technique was, therefore, considered to be insufficient according to the prescribed guidelines for 1 RM testing of youths (Faigenbaum et al., 2009). The back-squat test was performed by 21 senior females and 23 senior males.

##### The pull-up test

In this test, the participant was freely hanging from a barbell using an overhand grip at shoulder-width. The participant then performed a pull-up until the chin was horizontal to the barbell, followed by a lowering back to the starting position. The participant completed as many repetitions as possible without any break in between repetitions with the result being the maximum number of approved repetitions. A repetition was only approved if the gymnast had the chin at the level of the barbell, without kipping with the body and/or legs or changing the handgrip.

##### The dips test

This test was performed on two handles with the participant starting with straight arms at a shoulder-width position. The body was lowered until the back of the upper arm (i.e., triceps) was parallel to the floor, followed by an arm push-up back to the starting position. The participant completed as many repetitions as possible without any break in between repetitions with the result being the maximum number of approved repetitions.

##### The hanging sit-ups test

The test started with the participant hanging upside down from a bar in a position where the knees were fixed at a 90° knee joint angle using an inverted sit-up station. In the starting position, the gymnast hanged upside down with the whole back in contact with the backrest while holding a folded cotton band behind the neck where after the upper body was raised until the elbows touched the knees and returning to the starting position in a controlled movement (Harris et al., 2015). The participant performed as many repetitions as possible without any break in between repetitions with the result being the maximum number of approved repetitions.

All strength tests were conducted in the same order as reported in the text above and the participant had ~5 min of rest in between tests.

#### Jump, Sprint, and Endurance Tests

##### The SJ test

This test was performed as a maximum vertical jump from ~90° knee-joint flexion with a standstill of ~2–3 s, the feet placed at hip-width and the hands placed on the hips. The SJ test was approved when the participant, on the test leader's command and without countermovement, jumped as high as possible with a full extension of the hip- and knee joints and with take-off and landing at approximately the same spot on the floor [for more details see Markovic et al. (2004)].

##### The CMJ test

This test was performed as a maximum vertical jump starting from an upright standing position, with the feet placed at hip-width apart and with the hands placed on the hips. The jump was initiated with a quick squat to a self-selected knee joint angle that was followed by a maximal explosive jump. During the flight phase, the gymnast had to maintain a full extension of the hip- and knee joints, with take-off and landing at approximately the same spot on the floor [for more details see Markovic et al. (2004)].

##### The CMJa test

The test started with a quick squat to a self-selected knee-joint angle followed by an explosive jump while including a supporting arm swing. During the flight phase, the gymnast had to maintain a full extension of the hip and knee joints and the take-off and landing had to be at approximately the same spot on the floor. In comparison to a CMJ test, the CMJa test incorporates the coordination qualities of arms and legs (Cheng et al., 2008).

##### The DJ test

The test started with a step-out from an elevated platform with a drop onto the ground followed by an immediate maximal vertical response jump. The DJ was performed from two different heights, 20 and 40 cm, respectively. These heights were chosen based on previous training and testing routines. The participant was informed to have the shortest possible contact time and to jump as high as possible. Jump height (cm) and contact time (ms) were registered together with a reactive strength index (i.e., the dynamic explosive vertical jump capacity) calculated as the optimal fall height, i.e., the best result from the 20 or 40 cm platform heights multiplied by 10 and divided by contact time.

##### The 20-m linear sprint-run test

The participant started the test from a split stance position 50 cm behind the first photocell. Split times at 5 and 10 m, as well as the end time after 20 m, were recorded to the nearest 0.01 s. Prior to the test, the gymnast performed two 20-m sprint runs at ~80% of maximum speed, followed by ~5 min of passive rest before the test.

##### The 3,000-m run test

After ~5 min of warm-up running at a low-to-moderate intensity and a passive rest of ~5 min, a 3,000-m run test was completed. The participant was informed to complete the test as fast as possible. The test was performed on an indoor 200-m tartan track and was a modified version of the Cooper test (Cooper, 1968). The participant's time to complete the test was measured with a stopwatch and immediately after the test, the athlete rated his/her level of perceived exertion using the 6-20 rating of perceived exertion (RPE) scale (Borg, 1982). The 3,000-m run test was performed by 14 junior females, 19 senior females, 14 junior males, and 13 senior males.

All the included tests in the third testing session were conducted in the same order as reported in the text above. The third testing session took ~1.5 h to perform and the participants had ~10 min of rest in between jumps, sprints, and the 3,000-m run test.

### Statistics

A statistical power calculation was performed *a priori* using data from a previous study (Hansen et al., 2019), and unpublished data on Swedish junior and senior TeamGym athletes were used to assume the magnitude of the differences between sex and age-groups. For a power “cut-off” of 0.80 and an alpha level of 0.05, a minimum sample size of ~14 participants within each of the two age-groups was required (i.e., a minimum sample of 28 females and 28 males). The data was processed in Microsoft Excel 2019 (Microsoft Armonk, New York, USA) with statistical analyses performed in the Statistical Package for the Social Sciences **(**SPSS 21, IBM Corp, Redmond, Washington, USA) with the level of significance set at α = 0.05. The distribution of data was evaluated by visual inspection of Q–Q plots and histograms together with the Shapiro-Wilks analysis. Parametric tests were used for normally distributed data whereas non-parametric alternative tests were used for non-normally distributed data. Normally distributed data are presented as the mean ± standard deviation (SD) while non-normally distributed data, as well as ordinal data, are presented as the median and interquartile range (IQR). A two-way univariate ANOVA was used to analyze the main effects of sex and age group, as well as the interaction effect between sex and age group. In case of a significant interaction effect, a *post-hoc* independent *t*-test was used to compare juniors vs. seniors within each sex group with only significant results being reported. For skewed data or ordinal data, a Mann-Whitney U-test was used to analyze the main effects of sex and age group. In addition, an analysis of juniors and seniors within each sex group was performed with a Mann-Whitney U-test, with only significant results being reported. For the independent *t*-tests, the standardized mean difference (Hedges' *g* effect size [*H*_*g*_]) was computed according to the equations presented by Lakens (2013) and interpreted as small (*H*_*g*_ = 0.2), medium (*H*_*g*_ = 0.5), and large (*H*_*g*_ = 0.8). For the Mann-Whitney U-test, the *R* effect size was reported and calculated as the z value divided by the square root of the number of observations (i.e., *n*). The effect size for the ANOVA tests was presented as partial eta squared ($\eta_{p}^{2}$).

## Results

The anthropometrical characteristics are displayed in Table 1. The male athletes were, in comparison to the female athletes, 7% taller and 21% heavier which resulted in a body mass index (BMI) that was 5% higher. Senior compared to junior athletes (as both sexes combined) possessed a similar body height but were 10% heavier with a 7% higher BMI. This difference between age groups was mainly due to the substantially higher body mass (14%) and BMI (11%) for male senior vs. junior athletes as indicated by the significant interaction effects of sex on age group. In addition, significant differences in body mass and BMI between age groups were observed for the males with no such differences for the females. The training volume was significantly higher for seniors compared to juniors (15 vs. 13 h·week^−1^) with no significant differences between the sexes.

**Figure 2:**
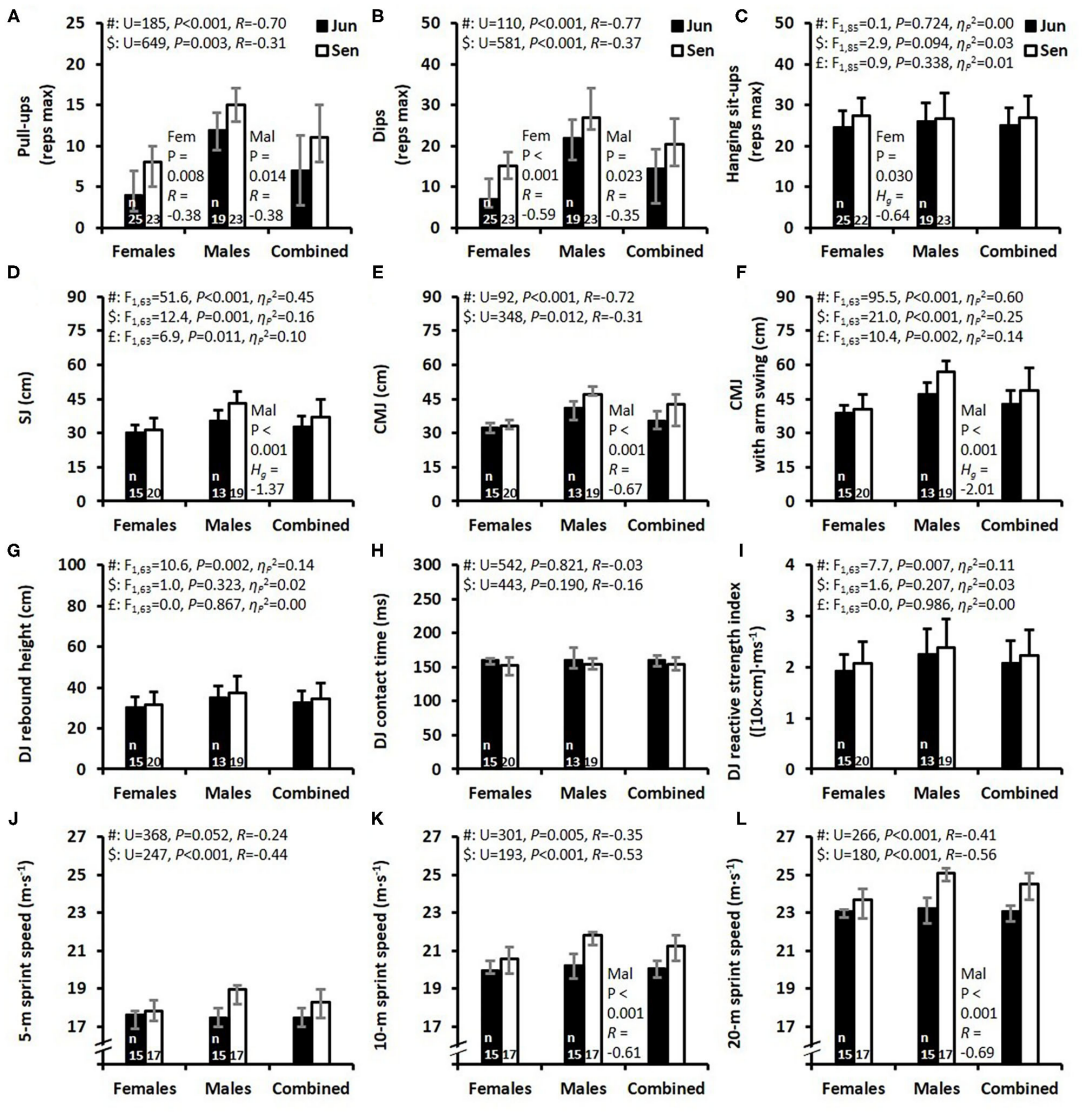
Results for the strength **(A–C)**, jumping **(D–I)**, and running sprint **(J-L)** performances presented as mean ± SD **(C,D,F,G,I)** and as median and interquartile range **(A,B,E,H,J–L)** for the junior (Jun) and senior (Sen) female and male athletes and the combined group of female and male athletes. Abbreviations: SJ, squat jump; CMJ, countermovement jump; DJ, drop jump; reps max, the maximum number of repetitions; Fem, females; Mal, males; Hg, Hedges' g effect size. F-values, P-values, and partial eta squared effect size ($\eta_{p}^{2}$), were obtained by a two-way ANOVA. #Main effect of sex. $Main effect of age group. £Main effect for interaction between sex and age group. In case of a significant interaction effect, a within-sex-group comparison of Jun vs. Sen was performed with an independent *t*-test **(D,F)** with significant results being reported. In **(A,B,E,H,J–L)**, *P*-values were obtained with a Mann-Whitney U-test presented together with an R effect size, and separate within-sex-group comparisons between juniors and seniors were performed using a Mann-Whitney *U*-test with significant results being reported. The numbers located at the bottom of each of the four bars indicate the number of participants (*n*) in the specific test.

The results from the strength tests are demonstrated in Figures 2A–C and show that the males were substantially stronger than the females in the pull-ups and dips exercises whereas there was no difference between the sexes for hanging sit-ups. Both female and male seniors were stronger than juniors in the pull-ups and dips exercises, while there was no effect of age group on the results for the hanging sit-ups. The back-squat strength ratio (i.e., the lifted weight divided by body mass) was 1.44 ± 0.17 and 1.79 ± 0.31 for the female and male seniors, respectively (*P* < 0.001, *H*_*g*_ = −1.35). The results for the SJ, CMJ, and CMJa tests are displayed in Figures 2D–F which show main effects of sex, age group, as well as an interaction effect between sex and age group with substantially larger differences between junior and senior males than between junior and senior females. The results from the DJ test are displayed in Figures 2G–I which show that the DJ rebound height and DJ reactive strength index were higher for the male athletes, with no difference between sexes in DJ contact time, and with no statistical main effect of age group for all DJ variables.

The average speeds for the first 5 and 10 m of the 20-m linear sprint-run test, as well as finishing time at 20 m, are shown in Figures 2J–L which demonstrates that males were significantly faster over 10 and 20 m compared to the females. The juniors combined were also significantly slower than the seniors at 5, 10, and 20 m. The male juniors were significantly slower than the male seniors at 10 and 20 m whereas the female juniors and seniors were similarly fast at 5, 10, and 20 m. The corresponding median (IQR) times at 5 m were 1.02 (1.01–1.07), 1.01 (0.98–1.04), 1.03 (1.00–1.06), 0.95 (0.94–0.99) s for the female juniors, female seniors, male juniors, and male seniors, respectively (main effect of sex: *U* = 368, *P* = 0.052, *R* = −0.24; main effect of age group: *U* = 247, *P* < 0.001, *R* = −0.44). The separate comparisons between juniors and seniors within each sex group demonstrated no significant differences. The corresponding times at 10 m were 1.80 (1.76–1.82), 1.75 (1.70–1.82), 1.78 (1.73–1.85), 1.65 (1.64–1.69) s for the female juniors, female seniors, male juniors, and male seniors, respectively (main effect of sex: *U* = 301, *P* = 0.005, *R* = −0.35; main effect of age group: *U* = 193, *P* < 0.001, *R* = −0.53). The separate comparisons between juniors and seniors within each sex group demonstrated significant differences for the male juniors vs. seniors (*P* < 0.001, *R* = −0.61) but with no difference for the female juniors vs. seniors. The corresponding finish times of the 20-m sprint run were 3.12 (3.11–3.17), 3.04 (2.97–3.17), 3.10 (3.03–3.21), 2.87 (2.84–2.92) s for the female juniors, female seniors, male juniors, and male seniors, respectively (main effect of sex: *U* = 266, *P* < 0.001, *R* = −0.41; main effect of age group: *U* = 180, *P* < 0.001, *R* = −0.56). Similar to the 10 m split time, the comparison between juniors and seniors within each sex group revealed a significant difference between male juniors and seniors (*P* < 0.001, *R* = −0.69), with no significant difference between the female junior and senior groups.

**Figure 3:**
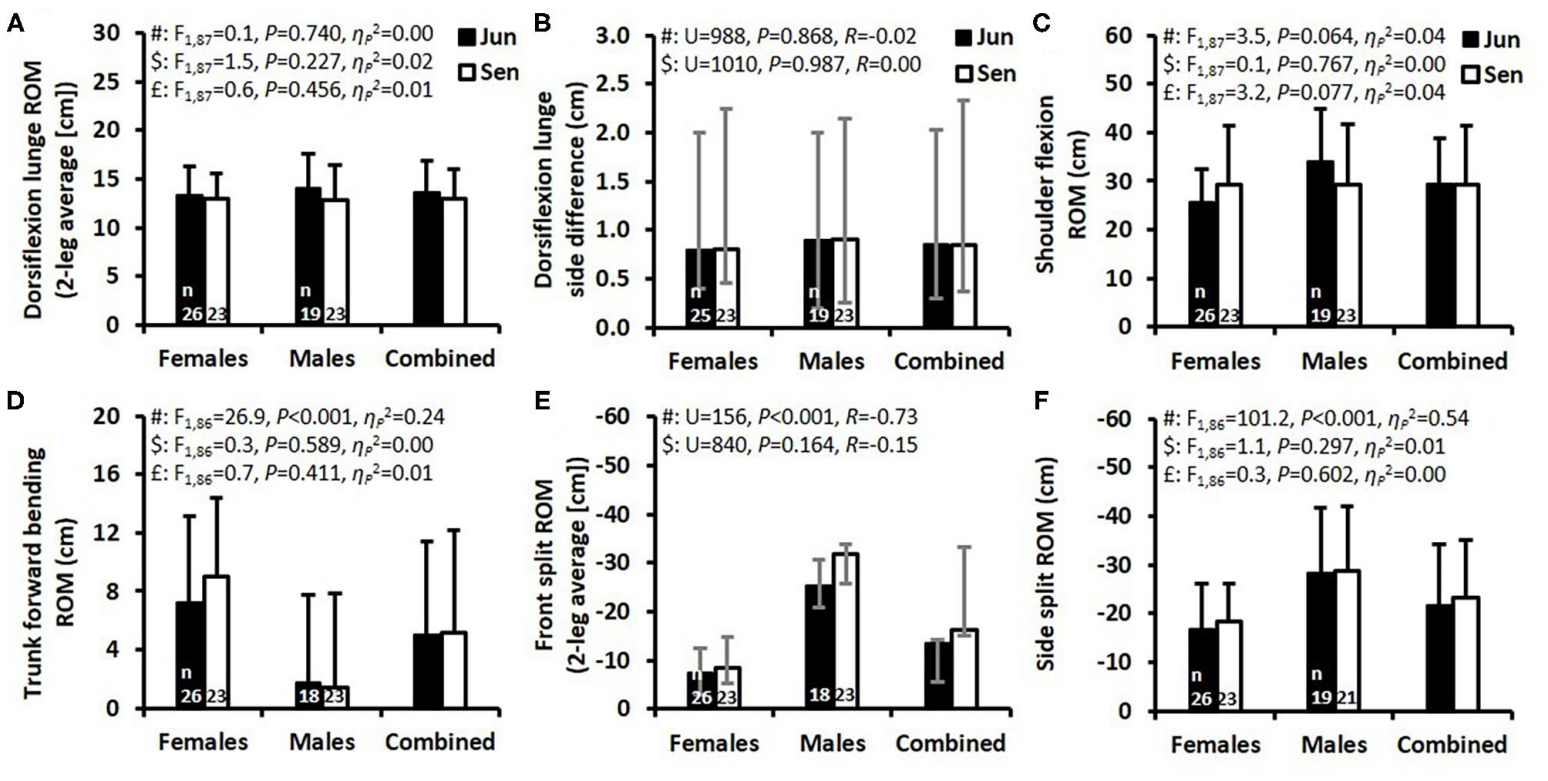
Flexibility test results presented as mean ± standard deviation (SD) **(A,C,D,F)** and as median and interquartile range **(B,E)** for the junior (Jun) and senior (Sen) female and male athletes and the combined group of female and male athletes. Abbreviations: ROM, range of motion; Hg, Hedges' g effect size. *F*-values, *P*-values, and partial eta squared effect size ($\eta_{p}^{2}$), were obtained by a two-way ANOVA. #Main effect of sex. $Main effect of age group. £Main effect for interaction between sex and age group. In **(B,E)**, *P*-values were obtained by a Mann-Whitney U-test presented together with an R effect size. The numbers located at the bottom of each of the four bars indicate the number of participants (*n*) in the specific test.

The flexibility test results that are shown in Figures 3A–F reveal that the females were significantly more flexible than the males in the trunk forward bending, front split, and side split tests (Figures 3D–F), but similarly flexible in the dorsiflexion lunge test (Figure 3A). However, the males scored higher flexibility in the shoulder flexion test than the females, but this difference was not significant (Figure 3C). All flexibility tests revealed no significant age group effects. The dorsiflexion lunge side difference (i.e., the difference between the most and least flexible legs) showed no main effects of sex or age group (Figure 3B). The difference (as median and IQR) between the most and least flexible legs during the front split was 5.0 (1.6–8.0), 5.0 (1.0–7.0), 3.0 (1.5–9.0), and 2.0 (1.0-6.8) cm for the female juniors, female seniors, male juniors, and male seniors, respectively (main effect of sex: *U* = 965, *P* = 0.607, *R* = −0.05; main effect of age group: *U* = 895, *P* = 0.265, *R* = −0.12). The separate comparisons between age groups within each sex revealed no significant differences.

The 3,000-m run test was completed in 15.5 ± 1.6, 14.8 ± 1.1, 13.5 ± 1.2, and 13.4 ± 1.2 min for the female juniors, female seniors, male juniors, and male seniors, respectively, with a significant main effect of sex, but no significant main effect of age group or interaction between sex and age group (sex: *F*_1,56_ = 25.7, *P* < 0.001, $\eta_{p}^{2}$ = 0.31; age group: *F*_1,56_ = 1.6, *P* = 0.206, $\eta_{p}^{2}$ = 0.03; interaction: *F*_1,56_ = 0.7, *P* = 0.395, $\eta_{p}^{2}$ = 0.01). The RPE immediately after the 3,000-m test was similar for female juniors (18 [16–19]), female seniors (18 [18–19]), male juniors (18 [17–19]), and male seniors (17 [16–18]) (main effect of sex: *U* = 329, *P* = 0.077, *R* = −0.23; main effect of age group: *U* = 407, *P* = 0.529, *R* = −0.08). Separate comparisons between juniors and seniors within each sex group revealed no significant differences in RPE. All the strength, jump, speed, endurance, and flexibility results can also be found in the Supplementary Tables.

## Discussion

This study aimed to describe the physical capacity of Swedish elite TeamGym athletes and to analyze sex differences, as well as compare junior with senior athletes. The results showed large sex differences in most of the physical performance parameters. The males were substantially stronger, jumped higher, sprinted faster, and had better endurance performance. The senior athletes performed, in comparison to the junior athletes, better in the pull-ups, dips, SJ, CMJ, CMJa, and 20-m sprint-run. However, sex-specific age-group comparisons for the SJ, CMJ, CMJa, and 20-m sprint-run tests revealed larger age-group effects in males than in females.

The results of sex differences observed in the current study align with previous findings that males are taller and heavier than females, and have greater maximal strength, vertical jump performance, sprinting speed, and endurance characteristics (Bishop et al., 1987; Bale et al., 1992; McMahon et al., 2017; Cardoso de Araújo et al., 2020). In comparison, females are significantly more flexible than males (Bale et al., 1992). To our knowledge, there is only one previous study that has analyzed the performance characteristics of elite TeamGym athletes (Hansen et al., 2019). We hypothesized, based on the latter study, that sex differences for the CMJ and the 20-m sprint run would be ~24% and ~9%, respectively. However, in the current study, the males jumped 34% higher than the females in the CMJ and the males completed the 20-m sprint run in 4% less time than the females. The substantially lower sex difference observed for the 20-m sprint, when compared to the CMJ, was unexpected, since moderate to very large correlations between 20-m sprint and CMJ performances have been observed previously in different groups of soccer players (Haugen et al., 2012). Haugen et al. (2012) also noticed that some equally fast athletes could vary by as much as 10–12 cm in a CMJ which indicates that different physiological and biomechanical factors are related to horizontal acceleration (i.e., sprinting) and vertical acceleration (i.e., jumping). However, as both factors appear to be important for TeamGym performance (Hansen et al., 2019; Schärer et al., 2019), athletes with better sprint skills may compensate for lower jump performance. The somewhat divergent findings observed for the sprint and jump performances in this study when compared to both our hypothesis and the study by Hansen et al. (2019) may be due to different athletic populations, where the current study assessed Swedish elite gymnasts recruited from the first selection of the national team, whereas Hansen et al. (2019) instead tested the final national team.

In the current study, the SJ, CMJ, and CMJa jump heights were lower for junior compared to senior athletes (for both sexes combined, on average ~12% lower) and the 20-m linear sprint-run was 5% slower for junior vs. senior athletes (3.13 s vs. 2.97 s). This was not surprising, as physical ability and sports performance are known to increase during adolescence (Handelsman, 2017), and therefore may partly explain such findings. However, as shown in Figure 2, these differences were mainly manifested in the male athletes as confirmed by the significant interaction effects between sex and age group. Separate age-group comparisons within each sex group revealed significant differences only between male juniors vs. seniors. This may be due to the substantial increase in muscle mass and strength observed during male adolescence, and the resultant divergence in physical ability and sports performance between the sexes, from puberty until ~20 years (Kraemer et al., 1989; Handelsman, 2017). For both age groups combined, the males jumped 29% higher in the SJ and 34% higher in both the CMJ and CMJa than the females. The somewhat larger sex difference for the two jumps that involved a countermovement indicates that the males were slightly more effective in utilizing the stretch-shortening cycle (Ziv and Lidor, 2010) and/or involving the hip extensor muscles (Lees et al., 2004) in the CMJ and CMJa than the females. In addition, the DJ height and DJ reactive strength index were both ~17% higher for the males vs. the females, which indicate, together with previous findings (Prieske et al., 2017), that males probably have better vertical leg stiffness and, thus, probably are better at utilizing the stretch-shortening cycle (Kipp et al., 2018).

Moreover, gymnasts are in general known for their high strength relative to body mass (Bale and Goodway, 1990), which was also confirmed in the current study as the 1 RM back-squat strength relative to body mass demonstrated higher values for both sexes compared to basketball players, and for males, a similar strength level to elite rugby players (Tanner and Gore, 2013). Although lower body strength is likely to be more important than upper body strength in TeamGym athletes, a sufficient level of upper-body strength is also likely to be important. In the current study, sex and age-group differences were found for the pull-ups and dips. However, the sex-specific differences between age groups were similar which was contrary to the results for the 20-m sprint run and the jump tests (SJ, CMJ, and CMJa). A potential explanation for this finding could be related to a combination of how TeamGym athletes train and respond to strength training as well as where they gain muscle during puberty. It can be noted that the male senior athletes were considerably heavier than the male junior athletes whereas no such difference was revealed in the female athletes.

The flexibility demands of TeamGym have increased since the latest update of the Code of Points in 2017 and the level of the floor routine elements has increased on a year-to-year basis, which puts higher demands on flexibility. The sport of gymnastics has always been characterized by high flexibility requirements, and indeed flexibility has been a critical component that separates gymnastics from other sports (Bale and Goodway, 1990). For TeamGym, the importance of this factor has substantially increased with the latest update of the Code of Points. The hip flexibility is likely to be of specific importance to TeamGym athletes, which is similar to the other gymnastic disciplines (Bale and Goodway, 1990). Since the current study showed males to be substantially less flexible than the females in the trunk forward bending, front split, and side split; male TeamGym athletes should potentially emphasize hip flexibility training more than their female counterparts.

The maximal aerobic power in artistic gymnastics has been studied for many years with maximal oxygen uptakes in a combined group of males and females reported to ~50 ml·kg^−1^·min^−1^ (Jemni et al., 2006). Therefore, it is logical to conclude that a gymnast needs a maximal oxygen uptake that is fairly similar to the value of a healthy female or male individual of the same age (Andersen et al., 1987; Aspenes et al., 2011). Although maximal oxygen uptake is probably not a direct key performance indicator in the performance of TeamGym, a sufficient level is probably important for optimal recovery from training and cardiovascular health (Carey et al., 2007; Aspenes et al., 2011). The 3,000-m run test result can be used to roughly evaluate the cardiovascular fitness of the gymnast since the more traditional 12-min run test introduced by Cooper (1968) has been used as a surrogate marker of cardiovascular fitness (Bandyopadhyay, 2015). As based on the 3,000-m run performances in the current study, average maximal oxygen uptakes for male and female TeamGym athletes were estimated as 42 and 49 ml·kg^−1^·min^−1^, respectively, and are fairly similar to values reported in previous studies for similar age groups from the general population (Andersen et al., 1987; Aspenes et al., 2011). However, the estimated values are likely to be slightly lower than the true values since the estimation was based on a 12-min distance (Bandyopadhyay, 2015) as calculated from the 3,000-m speeds; this is because the average completion time for the 3,000-m test was 14.4 min, i.e., higher than 12 min.

Compared to other elite team-sport athletes (futsal, handball, football, rugby), the TeamGym athletes in the current study showed ~5% higher vertical jump (SJ and CMJ) performances (Loturco et al., 2018) and seem to have higher sprinting and acceleration abilities compared to netball, tennis, cricket and basketball players, but similar sprinting and acceleration abilities as elite football players (Tanner and Gore, 2013). This data indicates that a high maximal sprinting speed for the run-up is important in TeamGym. Moreover, force impulse generation during the jump phase seems to be essential for successful performance. Gymnasts with high eccentric and concentric strength and explosive capacities can produce high force impulse and momentum; factors that likely contribute to more advanced acrobatics (French et al., 2004). It has been shown that resistance strength training programs in gymnastics can increase the force impulse in the CMJ and SJ tests, which enable gymnasts to jump higher and to perform better-scoring gymnastic acrobatic elements, mainly due to the increased flight time (French et al., 2004). The combination of an eccentric muscle action followed by fast concentric muscle action is described as the stretch-shortening cycle, which is a predictor of sprint capacity and high-speed movements in general (Komi, 2000). In this study, we used different jumps such as CMJ, CMJa, SJ, and DJ to evaluate the gymnast's potential to produce force via the lower body, which is very likely to influence the performance in TeamGym. In TeamGym, it is important to generate a high speed and thus forward momentum due to the long run-up in two of three apparatuses. In comparison, artistic gymnastic only requires a run-up for one of several apparatuses. Furthermore, a high run-up speed is correlated with the difficulty score in the vault in artistic gymnastics (Schärer et al., 2019). Due to this, a high run-up speed is likely to be more important to overall performance in TeamGym than in artistic gymnastics.

The sport of TeamGym has a lower focus on weight-bearing exercises compared to artistic gymnastics and involves the use of more rebounding equipment, where a longer contact time with the equipment enables a longer time for force-impulse generation. Such factors could describe, as compared to earlier findings, that our group of male TeamGym athletes was taller and heavier compared to athletes of similar age competing in artistic gymnastics (Jemni et al., 2006). The same logic applies to the female group in the current study since they were taller and heavier than artistic gymnasts of similar age (João and Filho, 2015). Based on our results, the amount of training hours per week seems to be substantially lower for TeamGym athletes compared to other gymnastic disciplines (Edouard et al., 2018).

The large sex differences for most of the test results presented in this study help to explain the fundamental differences in specific performance characteristics related to sex differences in TeamGym performance. The Code of Points has identical difficulty values for all elements in the routines for females and males during competitions, which may be an obstacle for females due to the large differences in physical performance. In comparison, females and males compete in different apparatuses and routines in artistic gymnastics and the Code of Points differs according to that. The participants in the current study were chosen for the national TeamGym team with selection criteria according to the gymnastic performance level. The same criteria were used for juniors and seniors to select the team, which could be an obstacle for juniors that hit puberty late, especially for male juniors. Since our results indicate that physical growth varies between sexes, physical vs. technical abilities should probably be considered differently by coaches/trainers in the pubertal male vs. female TeamGym athlete's training (Lloyd and Oliver, 2012).

### Limitations

In the current study, a cross-sectional study design was employed to provide descriptive information about the physical abilities of Swedish elite TeamGym athletes. Due to this, some findings should be interpreted with caution since such a design is inadequate for determining cause and effect relationships. For determining the effect of sex and age-group on the tested variables a prospective 5-year cohort study would have been more robust from a scientific perspective. However, such a study design was not possible to conduct on our target group of Swedish elite TeamGym athletes due to a multitude of factors. One main problem with such a design would have been related to the recruitment of athletes and potential drop-outs. Due to the cross-sectional study design, some results should be interpreted with caution, especially the interaction effects between sex and age-group. However, due to the sparse amount of published data on elite TeamGym athletes, the current study, based on a relatively big sample of elite athletes, provides important normative data for athletes, coaches, and trainers.

## Conclusions and Perspectives

Large sex differences were observed for most of the physical performance tests. Females had better flexibility than males, whereas males showed substantially better strength, jumping, speed, and endurance performance. The senior athletes performed better than the junior athletes in the pull-ups, dips, SJ, CMJ, CMJa, and 20-m linear sprint-run tests. However, the findings showed that there were small differences between junior and senior females with only significant differences observed for the strength tests. In contrast, between junior and senior males, significant differences were also revealed for jump, and sprint performances. These findings can be used for physical profiling by coaches when designing preparation strategies for athletic development for different age and sex groups. In addition, these results could be used for upcoming updates of the Code of Points ranking system for further development of the TeamGym discipline.

## Data Availability Statement

The raw data supporting the conclusions of this article will be made available by the authors, without undue reservation.

## Ethics Statement

The studies involving human participants were reviewed and approved by The Swedish Ethical Review Board. Written informed consent to participate in this study was provided by the participants' legal guardian/next of kin.

## Author Contributions

SH and EA designed the study, interpreted the results, and wrote the first draft. SH collected data. EA performed the statistical analysis and the presentation of the results. Both authors revised the manuscript and approve the final version to be published and agree to be accountable for all aspects of the work.

## Conflict of Interest

The authors declare that the research was conducted in the absence of any commercial or financial relationships that could be construed as a potential conflict of interest.

## Footnotes

^1^Fysprofilen (2016). Available at: https://fysprofilen.se/sv/default.aspx?PageID=1142.

## References

[B1] Andersen L. B. Henckel P. Saltin B. (1987). Maximal oxygen uptake in Danish adolescents 16-19 years of age. Eur. J. Appl. Physiol. Occup. Physiol. 56, 74–82. 10.1007/BF00696380 3104033

[B2] Askow A. T. Merrigan J. J. Neddo J. M. Oliver J. M. Stone J. D. Jagim A. R.. (2019). Effect of strength on velocity and power during back squat exercise in resistance-trained men and women. J. Strength Cond. Res. 33, 1–7. 10.1519/JSC.0000000000002968 30431534

[B3] Aspenes S. T. Nilsen T. I. Skaug E. A. Bertheussen G. F. Ellingsen Ø. Vatten L.. (2011). Peak oxygen uptake and cardiovascular risk factors in 4631 healthy women and men. Med. Sci. Sports Exerc. 43, 1465–1473. 10.1249/MSS.0b013e31820ca81c 21228724

[B4] Bale P. Goodway J. (1990). Performance variables associated with the competitive gymnast. Sports Med. 10, 139–145. 10.2165/00007256-199010030-00001 2237031

[B5] Bale P. Mayhew J. L. Piper F. C. Ball T. E. Willman M. K. (1992). Biological and performance variables in relation to age in male and female adolescent athletes. J. Sports Med. Phys. Fitness 32, 142–148. 1434582

[B6] Bandyopadhyay A. (2015). Validity of Cooper's 12-minute run test for estimation of maximum oxygen uptake in male university students. Biol. Sport 32, 59–63. 10.5604/20831862.1127283 25729151 PMC4314605

[B7] Bencke J. Damsgaard R. Saekmose A. Jørgensen P. Jørgensen K. Klausen K. (2002). Anaerobic power and muscle strength characteristics of 11 years old elite and non-elite boys and girls from gymnastics, team handball, tennis and swimming. Scand. J. Med. Sci. Sports 12, 171–178. 10.1034/j.1600-0838.2002.01128.x 12135450

[B8] Bishop P. Cureton K. Collins M. (1987). Sex difference in muscular strength in equally-trained men and women. Ergonomics 30, 675–687. 10.1080/00140138708969760 3608972

[B9] Borg G. A. (1982). Psychophysical bases of perceived exertion. Med. Sci. Sports Exerc. 14, 377–381. 10.1249/00005768-198205000-00012 7154893

[B10] Cardoso de Araújo M. Baumgart C. Jansen C. T. Freiwald J. Hoppe M. W. (2020). Sex differences in physical capacities of German Bundesliga soccer players. J. Strength Cond. Res. 34, 2329–2337. 10.1519/JSC.0000000000002662 29927885

[B11] Carey D. G. Drake M. M. Pliego G. J. Raymond R. L. (2007). Do hockey players need aerobic fitness? Relation between VO2max and fatigue during high-intensity intermittent ice skating. J. Strength Cond. Res. 21, 963–966. 10.1519/00124278-200708000-00051 17685680

[B12] Carlsson M. Carlsson T. Hammarström D. Tiivel T. Malm C. Tonkonogi M. (2012). Validation of physiological tests in relation to competitive performances in elite male distance cross-country skiing. J. Strength Cond. Res. 26, 1496–1504. 10.1519/JSC.0b013e318231a799 22614140

[B13] Cheng K. B. Wang C. H. Chen H. C. Wu C. D. Chiu H. T. (2008). The mechanisms that enable arm motion to enhance vertical jump performance-a simulation study. J. Biomech. 41, 1847–1854. 10.1016/j.jbiomech.2008.04.004 18514208

[B14] Cooper K. H. (1968). A means of assessing maximal oxygen intake. Correlation between field and treadmill testing. JAMA 203, 201–204. 10.1001/jama.1968.03140030033008 5694044

[B15] Edouard P. Steffen K. Junge A. Leglise M. Soligard T. Engebretsen L. (2018). Gymnastics injury incidence during the 2008, 2012 and 2016 olympic Games: analysis of prospectively collected surveillance data from 963 registered gymnasts during Olympic Games. Br. J. Sports Med. 52, 475–481. 10.1136/bjsports-2017-097972 29032364

[B16] Faigenbaum A. D. Kraemer W. J. Blimkie C. J. Jeffreys I. Micheli L. J. Nitka M.. (2009). Youth resistance training: updated position statement paper from the national strength and conditioning association. J. Strength Cond. Res. 23, S60–79. 10.1519/JSC.0b013e31819df407 19620931

[B17] Fink H. Hofmann D. Ortiz López L. (2015). Age Group Development and Competition Program for Women's Artistic Gymnastics. Federation Internationale de Gymnastique (FIG).

[B18] French D. N. Gómez A. L. Volek J. S. Rubin M. R. Ratamess N. A. Sharman M. J.. (2004). Longitudinal tracking of muscular power changes of NCAA Division I collegiate women gymnasts. J. Strength Cond. Res. 18, 101–107. 10.1519/00124278-200402000-00015 14971975

[B19] Glatthorn J. F. Gouge S. Nussbaumer S. Stauffacher S. Impellizzeri F. M. Maffiuletti N. A. (2011). Validity and reliability of Optojump photoelectric cells for estimating vertical jump height. J. Strength Cond. Res. 25, 556–560. 10.1519/JSC.0b013e3181ccb18d 20647944

[B20] Handelsman D. J. (2017). Sex differences in athletic performance emerge coinciding with the onset of male puberty. Clin. Endocrinol. 87, 68–72. 10.1111/cen.13350 28397355

[B21] Handelsman D. J. Hirschberg A. L. Bermon S. (2018). Circulating testosterone as the hormonal basis of sex differences in athletic performance. Endocr. Rev. 39, 803–829. 10.1210/er.2018-00020 30010735 PMC6391653

[B22] Hansen O. H. Hvid L. G. Aagaard P. Jensen K. (2019). Mechanical lower limb muscle function and its association with performance in elite team Gymnasts. Sci. Gymnastics J. 11, 163–174.

[B23] Harringe M. L. Lindblad S. Werner S. (2004). Do team gymnasts compete in spite of symptoms from an injury? Br. J. Sports Med. 38, 398–401. 10.1136/bjsm.2002.001990 15273170 PMC1724898

[B24] Harringe M. L. Renström P. Werner S. (2007). Injury incidence, mechanism and diagnosis in top-level teamgym: a prospective study conducted over one season. Scand. J. Med. Sci. Sports 17, 115–119. 10.1111/j.1600-0838.2006.00546.x 17394471

[B25] Harris A. Gundersen H. Mørk-Andreassen P. Thun E. Bjorvatn B. Pallesen S. (2015). Restricted use of electronic media, sleep, performance, and mood in high school athletes–a randomized trial. Sleep Health 1, 314–321. 10.1016/j.sleh.2015.09.011 29073407

[B26] Haugen T. A. Tønnessen E. Seiler S. (2012). Speed and countermovement-jump characteristics of elite female soccer players, 1995-2010. Int. J. Sports Physiol. Perform. 7, 340–349. 10.1123/ijspp.7.4.340 22645175

[B27] Hughes K. Lemmetty H. Tybjerg-Pedersen E. Sjöstrand P. Boemann C. Dvoracek R.. (2010). Code of Points TeamGym, 2009, Including Revision A, August 2010. Retrieved from: http://www.ueg-gymnastics.com/members-data (accessed November 13, 2011).

[B28] Jemni M. Sands W. A. Friemel F. Stone M. H. Cooke C. B. (2006). Any effect of gymnastics training on upper-body and lower-body aerobic and power components in national and international male gymnasts? J. Strength Cond. Res. 20, 899–907. 10.1519/00124278-200611000-00029 17149990

[B29] João A. F. Filho J. F. (2015). Somatotype and body composition of elite Brazilian gymnasts. Sci. Gymnastics J. 7, 45–54.

[B30] Kipp K. Kiely M. T. Giordanelli M. D. Malloy P. J. Geiser C. F. (2018). Biomechanical determinants of the reactive strength index during drop jumps. Int. J. Sports Physiol. Perform. 13, 44–49. 10.1123/ijspp.2017-0021 28422586

[B31] Komi P. V. (2000). Stretch-shortening cycle: a powerful model to study normal and fatigued muscle. J. Biomech. 33, 1197–1206. 10.1016/S0021-9290(00)00064-6 10899328

[B32] Kraemer W. J. Fry A. C. Frykman P. N. Conroy B. Hoffman J. R. (1989). Resistance training and youth. Pediatr. Exerc. Sci. 1, 336–350. 10.1123/pes.1.4.336 36949594

[B33] Lakens D. (2013). Calculating and reporting effect sizes to facilitate cumulative science: a practical primer for t-tests and ANOVAs. Front. Psychol. 4:863. 10.3389/fpsyg.2013.00863 24324449 PMC3840331

[B34] Lees A. Vanrenterghem J. De Clercq D. (2004). The maximal and submaximal vertical jump: implications for strength and conditioning. J. Strength Cond. Res. 18, 787–791. 10.1519/00124278-200411000-00018 15574084

[B35] Levinger I. Goodman C. Hare D. L. Jerums G. Toia D. Selig S. (2009). The reliability of the 1RM strength test for untrained middle-aged individuals. J. Sci. Med. Sport 12, 310–316. 10.1016/j.jsams.2007.10.007 18078784

[B36] Lloyd R. S. Oliver J. L. (2012). The youth physical development model: a new approach to long-term athletic development. Strength Cond. J. 34, 61–72. 10.1519/SSC.0b013e31825760ea25486295

[B37] Loturco I. Suchomel T. James L. P. Bishop C. Abad C. C. C. Pereira L. A.. (2018). Selective influences of maximum dynamic strength and bar-power output on team sports performance: a comprehensive study of four different disciplines. Front. Physiol. 9:1820. 10.3389/fphys.2018.01820 30618830 PMC6304672

[B38] Lund S. S. Myklebust G. (2011). High injury incidence in TeamGym competition: a prospective cohort study. Scand. J. Med. Sci. Sports 21, e439–e444. 10.1111/j.1600-0838.2011.01362.x 21812827

[B39] Markovic G. Dizdar D. Jukic I. Cardinale M. (2004). Reliability and factorial validity of squat and countermovement jump tests. J. Strength Cond. Res. 18, 551–555. 10.1519/00124278-200408000-00028 15320660

[B40] McMahon J. J. Rej S. J. E. Comfort P. (2017). Sex differences in countermovement jump phase characteristics. Sports 5:8. 10.3390/sports5010008 29910368 PMC5969005

[B41] Nuell S. Illera-Domínguez V. Carmona G. Alomar X. Padullés J. M. Lloret M.. (2019). Sex differences in thigh muscle volumes, sprint performance and mechanical properties in national-level sprinters. PLoS ONE 14:e0224862. 10.1371/journal.pone.0224862 31689336 PMC6830821

[B42] Prieske O. Demps M. Lesinski M. Granacher U. (2017). Combined effects of fatigue and surface instability on jump biomechanics in elite athletes. Int. J. Sports Med. 38, 781–790. 10.1055/s-0043-111894 28768338

[B43] Schärer C. Lehmann T. Naundorf F. Taube W. Hübner K. (2019). The faster, the better? Relationships between run-up speed, the degree of difficulty (D-score), height and length of flight on vault in artistic gymnastics. PLoS ONE 14:e0213310. 10.1371/journal.pone.0213310 30845256 PMC6405201

[B44] Sjöstrand P. Lemmetty H. Hughes K. Tranckle P. Gryga P. Jónsdóttir S.. (2017). European Gymnastics (former Union Européenne de Gymnastique), Avenue de la Gare 12, 1003 Lausanne, Switzerland. Available online at: www.europeangymnastics.com

[B45] Sleeper M. D. Kenyon L. K. Casey E. (2012). Measuring fitness in female gymnasts: the gymnastics functional measurement tool. Int. J. Sports Phys. Ther. 7, 124–138. 22530187 PMC3325636

[B46] Sleeper M. D. Kenyon L. K. Elliott J. M. Cheng M. S. (2016). Measuring sport-specific physical abilities in male gymnasts: the men's gymnastics functional measurement tool. Int. J. Sports Phys. Ther. 11, 1082–1100. 27999723 PMC5159633

[B47] Tanner R. Gore C. (2013). Physiological Tests for Elite Athletes. 2nd Ed. Champaign, IL: Human Kinetics.

[B48] Ziv G. Lidor R. (2010). Vertical jump in female and male basketball players–a review of observational and experimental studies. J. Sci. Med. Sport 13, 332–339. 10.1016/j.jsams.2009.02.009 19443269

