# Supplementary material for: Sex and Age-Group Differences in Strength, Jump, Speed, Flexibility, and Endurance Performances of Swedish Elite Gymnasts Competing in TeamGym
Source: Front Sports Act Living. 2021 May 13;3:653503. doi: 10.3389/fspor.2021.653503 (PMC8158296; doi:10.3389/fspor.2021.653503)
Supplement: Supplementary file 1 [file Data_Sheet_1.docx]

Supplementary Material

# Supplementary Tables

**Table S1.** Results for the strength, jump, sprint, and endurance performances presented as mean ± standard deviation, for normally distributed data, or as median and interquartile range (in parenthesis), for non-normally distributed data, and the number of participants (n) for the junior (J) and senior (S) female and male TeamGym athletes.

|  |  | Females | Males |
| --- | --- | --- | --- |
| Back squat relative strength | J | - | - |
| ratio (LW∙BM^-1^) | S | 1.4 ± 0.2 (n=21) | 1.8 ± 0.3 (n=23) |
| Pull-ups (reps max) | J | 4.5 ± 3.3 (n=25) | 12.5 ± 4.6 (n=19) |
|  | S | 7.5 ± 3.8 (n=23) | 15.7 ± 5.4 (n=23) |
| Dips (reps max) | J | 7 (5-12) (n=25) | 22 (17-27) (n=19) |
|  | S | 15 (12-19) (n=23) | 27 (24-34) (n=23) |
| Hanging sit-ups (reps max) | J | 24.6 ± 4.1 (n=25) | 25.9 ± 4.5 (n=19) |
|  | S | 27.3 ± 4.3 (n=22) | 26.7 ± 6.1 (n=23) |
| SJ (cm) | J | 30.3 ± 3.1 (n=15) | 35.6 ± 4.6 (n=13) |
|  | S | 31.3 ± 5.1 (n=20) | 42.9 ± 5.5 (n=19) |
| CMJ (cm) | J | 32.3 (30.0-34.5) (n=15) | 41.0 (35.8-43.9) (n=13) |
|  | S | 33.1 (32.0-35.9) (n=20) | 46.8 (46.5-50.3) (n=19) |
| CMJa (cm) | J | 38.8 ± 3.6 (n=15) | 47.2 ± 4.9 (n=13) |
|  | S | 40.6 ± 6.4 (n=20) | 57.1 ± 4.8 (n=19) |
| DJ rebound height (cm) | J | 30.5 ± 4.9 (n=15) | 35.4 ± 5.1 (n=13) |
|  | S | 31.8 ± 6.2 (n=20) | 37.2 ± 8.2 (n=19) |
| DJ contact time (ms) | J | 158.3 ± 11.0 (n=15) | 161.9 ± 29.2 (n=13) |
|  | S | 154.5 ± 19.2 (n=20) | 156.5 ± 14.1 (n=19) |
| DJ RSI ([10×cm]∙ms^-1^) | J | 1.9 ± 0.3 (n=15) | 2.2 ± 0.5 (n=13) |
|  | S | 2.1 ± 0.4 (n=20) | 2.4 ± 0.5 (n=19) |
| 5-m sprint time (s) | J | 1.04 ± 0.05 (n=15) | 0.99 ± 0.04 (n=13) |
|  | S | 1.05 ± 0.05 (n=19) | 0.97 ± 0.05 (n=17) |
| 10-m sprint time (s) | J | 1.80 ± 0.06 (n=15) | 1.74 ± 0.06 (n=13) |
|  | S | 1.80 ± 0.07 (n=19) | 1.66 ± 0.05 (n=17) |
| 20-m sprint time (s) | J | 3.15 ± 0.08 (n=15) | 3.02 ± 0.10 (n=13) |
|  | S | 3.13 ± 0.11 (n=19) | 2.88 ± 0.07 (n=17) |
| 3000-m run time (min) | J | 15.5 ± 1.6 (n=14) | 13.5 ± 1.2 (n=14) |
|  | S | 14.8 ± 1.1 (n=19) | 13.4 ± 1.2 (n=13) |

Abbreviations: SJ, squat jump; CMJ, countermovement jump; CMJ, countermovement jump with arm swing; DJ, drop jump; RSI, reactive strength index; LW, lifted weight (kg); BM, body mass (kg); reps max, maximum number of repetitions.

**Table S2.** Flexibility test results presented as mean ± standard deviation, for normally distributed data, or as median and interquartile range (in parenthesis), for non-normally distributed data, and the number of participants (n) for the junior (J) and senior (S) female and male TeamGym athletes.

|  |  | Females | Males |
| --- | --- | --- | --- |
| One-leg dorsiflexion | J | 13.3 ± 2.9 (n=26) | 14.1 ± 3.5 (n=19) |
| ROM (avg [cm]) | S | 13.0 ± 2.5 (n=23) | 12.8 ± 3.6 (n=23) |
| One-leg dorsiflexion | J | 0.8 (0.4-1.2) (n=25) | 0.9 (0.7-1.1) (n=19) |
| side difference (cm) | S | 0.7 (0.4-1.5) (n=23) | 1.0 (0.6-1.3) (n=23) |
| Shoulder flexion | J | 25.8 ± 6.6 (n=26) | 34.1 ± 10.9 (n=19) |
| ROM (cm) | S | 29.2 ± 12.1 (n=23) | 29.3 ± 12.5 (n=23) |
| Trunk forward | J | 7.3 ± 5.9 (n=26) | 1.8 ± 6.0 (n=18) |
| bending ROM (cm) | S | 9.0 ± 5.4 (n=23) | 1.4 ± 6.4 (n=23) |
| Front split ROM | J | -7.4 (-12.4 to -3.1) (n=26) | -25.4 (-30.6 to -21.0) (n=18) |
| (avg [cm]) | S | -8.5 (-14.8 to -5.4) (n=23) | -31.8 (-33.8 to -25.9) (n=23) |
| Front split | J | 5.0 (1.6-8.0) (n=26) | 3.0 (1.5-9.0) (n=18) |
| side difference (cm) | S | 5.0 (1.0-7.0) (n=23) | 2.0 (1.0-6.8) (n=23) |
| Side split | J | -16.8 ± 9.3 (n=26) | -28.4 ± 13.4 (n=19) |
| ROM (cm) | S | -18.3 ± 7.7 (n=23) | -28.8 ± 13.2 (n=21) |

Abbreviations: ROM, range of motion; avg, average of left and right leg.
